# Spontaneous α Brain Dynamics Track the Episodic “When”

**DOI:** 10.1523/JNEUROSCI.0816-23.2023

**Published:** 2023-10-25

**Authors:** Leila Azizi, Ignacio Polti, Virginie van Wassenhove

**Affiliations:** ^1^Cognitive Neuroimaging Unit, NeuroSpin, Commissariat à l'énergie atomique et aux énergies alternatives, Institut National de la Santé et de la Recherche Médicale, Université Paris-Saclay, Gif/Yvette 91191, France; ^2^Kavli Institute for Systems Neuroscience, Norwegian University of Science and Technology, Trondheim, Norway 7030; ^3^Department of Psychology, Max Planck Institute for Human Cognitive and Brain Sciences, Leipzig, Germany D-04103

**Keywords:** burst, neural oscillations, nonstationarity, passage of time, retrospective duration, time perception

## Abstract

Across species, neurons track time over the course of seconds to minutes, which may feed the sense of time passing. Here, we asked whether neural signatures of time-tracking could be found in humans. Participants stayed quietly awake for a few minutes while being recorded with magnetoencephalography (MEG). They were unaware they would be asked how long the recording lasted (retrospective time) or instructed beforehand to estimate how long it will last (prospective timing). At rest, rhythmic brain activity is nonstationary and displays bursts of activity in the alpha range (α: 7–14 Hz). When participants were not instructed to attend to time, the relative duration of α bursts linearly predicted individuals' retrospective estimates of how long their quiet wakefulness lasted. The relative duration of α bursts was a better predictor than α power or burst amplitude. No other rhythmic or arrhythmic activity predicted retrospective duration. However, when participants timed prospectively, the relative duration of α bursts failed to predict their duration estimates. Consistent with this, the amount of α bursts was discriminant between prospective and retrospective timing. Last, with a control experiment, we demonstrate that the relation between α bursts and retrospective time is preserved even when participants are engaged in a visual counting task. Thus, at the time scale of minutes, we report that the relative time of spontaneous α burstiness predicts conscious retrospective time. We conclude that in the absence of overt attention to time, α bursts embody discrete states of awareness constitutive of episodic timing.

**SIGNIFICANCE STATEMENT** The feeling that time passes is a core component of consciousness and episodic memory. A century ago, brain rhythms called “α” were hypothesized to embody an internal clock. However, rhythmic brain activity is nonstationary and displays on-and-off oscillatory bursts, which would serve irregular ticks to the hypothetical clock. Here, we discovered that in a given lapse of time, the relative bursting time of α rhythms is a good indicator of how much time an individual will report to have elapsed. Remarkably, this relation only holds true when the individual does not attend to time and vanishes when attending to it. Our observations suggest that at the scale of minutes, α brain activity tracks episodic time.

## Introduction

Brain rhythms in the α range (α: 7–14 Hz) are canonical markers of the level of consciousness in humans (Berger, 1935; Fell et al., 2010; Klimesch, 2012). They represent neural synchronization in spontaneous fluctuations with a period of ∼100 ms generated from a variety of neural sources (Steriade et al., 1990; Steriade, 1999; Raichle, 2015; Halgren et al., 2019; Higgins et al., 2021). Because of their omnipresence at rest, α rhythms were postulated to be the internal clock supporting one's awareness of the passage of time (Treisman, 1963, 1984; Kononowicz and van Wassenhove, 2016; van Wassenhove et al., 2019). To date however, whether spontaneous oscillations can predict an individual's experience of the passage of time at the scale of minutes remains unverified (Kononowicz and van Wassenhove, 2016; van Wassenhove et al., 2019). Here, we re-assess the original α clock hypothesis and ask whether bursts of spontaneous α activity keep track of time. This question was motivated by known nonstationarities in brain rhythms challenging the role of neural oscillations in cognition (Steriade et al., 1990; Cole and Voytek, 2017; van Ede et al., 2018), recent description of time cells with long and diverse periods (Pastalkova et al., 2008; MacDonald et al., 2011; Kraus et al., 2015; Issa et al., 2020; Umbach et al., 2020; Aghajan et al., 2022; Cogno et al., 2022; Tsao et al., 2022), and the identification of paradigmatic shortcomings in earlier work.

The clock hypothesis posits that in the absence of external sensory inputs, endogenous oscillatory activity (the pacemaker of the hypothesized internal clock) predicts an individual's estimation of elapsing time (Hoagland, 1935; Treisman, 1963). The clock hypothesis was built on the intuition that biological tick signals are steady and reliable enough to keep count of time units, like mechanical clocks periodically mark the passing of seconds. In its neurobiological implementation, the tick of the internal clock would be isomorphic to the period of spontaneous α neural oscillations. Hence, the α clock hypothesis made the assumption that neural oscillations are stationary, continuous and steadily persistent over time, i.e., can instantiate pacemaker-like rhythmic activity (e.g., Miall, 1989; Gibbon and Church, 1990; Buhusi and Meck, 2005; Gu et al., 2015; Kononowicz and van Wassenhove, 2016). Under this assumption, measuring an individual's α peak frequency (iAPF) would be equivalent to assessing the rate of change in time units, a metrical basis for the estimation of time. In a series of experiments, Michel Treisman, the instigator of the α clock hypothesis, dismissed this idea (Treisman, 1984): he observed that α oscillations did not behave like regular pacemakers. As is now acknowledged, spontaneous brain rhythms display nonstationarities with “up and down states” of bursting activity over time (Steriade et al., 1990; Jones, 2016; Sherman et al., 2016; Cole and Voytek, 2017; Shin et al., 2017).

Here, we wished to characterize spontaneous α brain activity while participants, quietly awake, were unaware they would have to report how much time had just elapsed. In human research, this can be done using a retrospective timing task, in which participants do not know in advance that time is the experimental factor of interest. Retrospective timing tasks engage episodic memory processes (Michon, 1975; Hicks et al., 1976; Block, 1985), and are under-studied for two reasons. First, retrospective timing is most relevant and ecologically valid over longer time scales (seconds to minutes and hours) but this time scale prevents collecting many trials within a single experiment (Grondin, 2010; Chaumon et al., 2022; Balcı et al., 2023). Second, and most importantly, a conservative retrospective task tests a single trial per participant to prevent attentional re-orientation to time, which would defeat the purpose of the task. With both these conditions fulfilled, retrospective timing emulates life events, mostly single shot experiences in our episodic landscape, and engage memory mechanisms (Michon, 1975; Hicks et al., 1976; Block, 1985). Here, we contend that this stringent approach allows addressing the basic building block for the automatic coding of the passage of time, at the minute-scale, in a manner very close to real life situations and comparable to interspecies approaches.

Our study is unique for several theoretical and empirical reasons. Neuroimaging studies mostly focus on prospective time, when participants covertly or overtly pay attention to time. Here, our interest is how the brain codes elapsing time when participants do not a priori adopt a cognitive strategy to estimate it. Timing tasks mostly focus on how the temporal statistics of external sensory events are attended to, predicted, analyzed, or categorized; here, we ask how elapsing time in the absence of sensory stimulation is encoded. Thus, we assess how endogenous processes during resting-state (Raichle, 2015) contribute to the retrospective sense of time constitutive of the episodic “when” (Friedman, 1993; Sugar and Moser, 2019; Buhusi, 2020). Last, timing tasks typically address short time-scales that are below a few seconds with a repeated number of trials time-locked to stimulations (Busch and VanRullen, 2010; Hanslmayr et al., 2011; Chakravarthi and VanRullen, 2012; Jensen et al., 2012; Landau and Fries, 2012; Grabot et al., 2017, 2021; Nobre and Van Ede, 2018; Mioni et al., 2020). Under these experimental conditions, the assumption that α oscillations are stationary is a fair approximation of the signals. At the longer episodic time scales investigated here, the assumption of stationarity is clearly violated, which motivates the novel characterization of spontaneous α activity we explored in this study.

## Materials and Methods

### Participants

All participants provided a written informed consent in accordance with the Ethics Committee on Human Research at NeuroSpin (Gif-sur-Yvette, France) and in conformity with the Declaration of Helsinki (2018). A total of 63 right-handed participants (27 males; age = 27 years old, ±6 years) were recruited for the first study. All had normal or corrected-to-normal vision and were naive as to the purpose of the study. None declared neurologic or psychiatric disorders, and none were under medical treatment. Seven participants were excluded a priori from the magnetoencephalographic (MEG) analysis: one participant showed an extreme time estimation (above the interquartile range), four participants showed nonrecoverable noisy MEG data and two participants did not comply with the task. Hence, a total of 56 participants (22 males; age = 27 years old, ±6 years) were analyzed in the retrospective time task.

Out of the 56 participants tested in the retrospective time task, a subgroup of 25 participants performed a prospective duration estimation task: one participant was excluded from the analysis because of an extreme estimation (above the interquartile range) yielding a final sample for the prospective group of 24 participants (11 males; age = 26 years old, ±5 years).

A new group of 26 right-handed participants (12 males; age = 24 years old, ±5 years) were recruited for the visual counting experiment. Three participants were excluded a priori from the MEG analysis: one participant showed an extreme time estimation (above the interquartile range), two participants showed nonrecoverable noisy MEG data. Hence, a total of 23 participants (11 males; age = 25 years old, ±5 years) were analyzed in the retrospective dual-task.

### Experimental design

**Figure 1.**
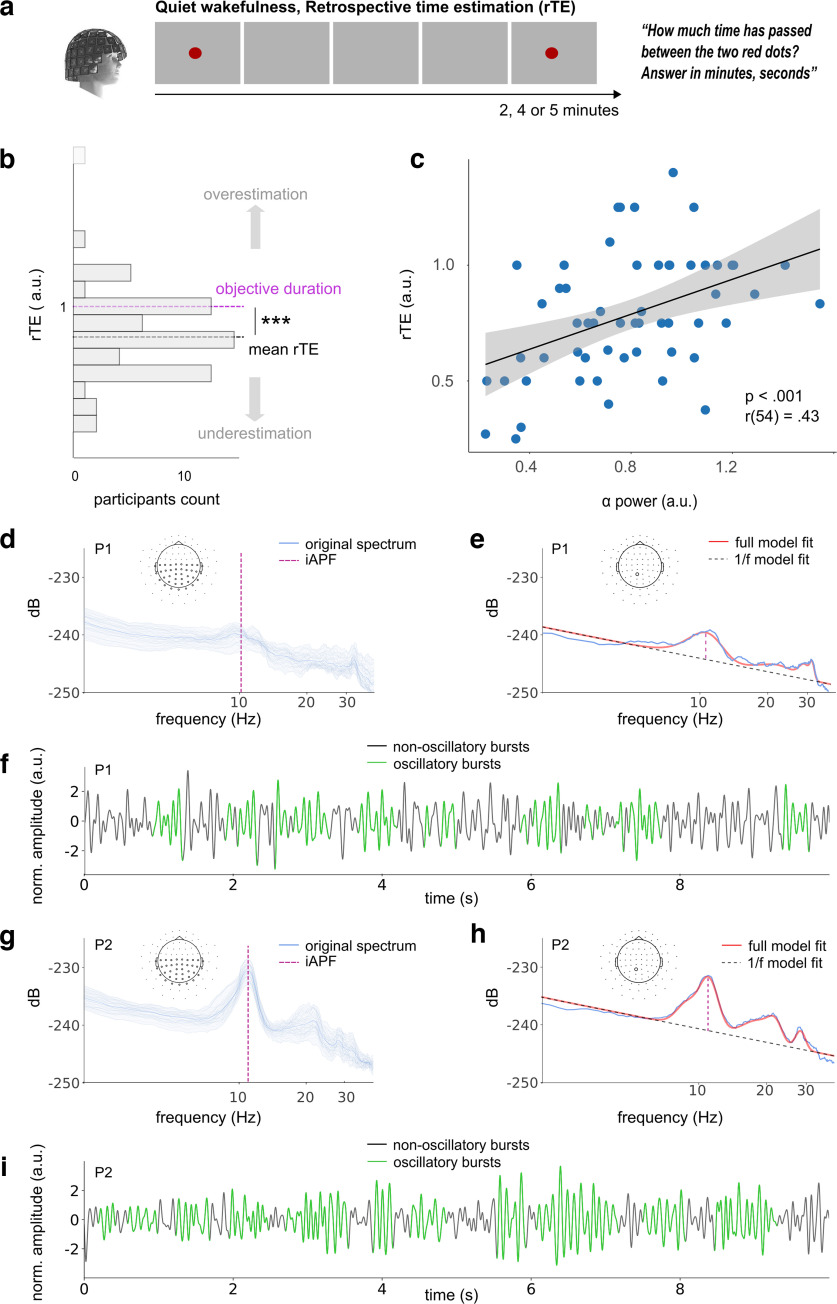
Retrospective time estimates. ***a***, In the retrospective timing task, participants (*N* = 56) stayed in quiet wakefulness during an MEG recording that could last 2, 4, or 5 min. Participants received no additional instructions. At the end of the MEG recording, they were asked to estimate as best they could the duration that elapsed between the two red dots, marking the beginning and end of the recording. ***b***, Distribution of the relative retrospective time estimates (rTE) across participants (*N* = 56). The dashed purple line delineates equality between subjective (rTE) and objective (clock) duration. The dashed gray line indicates the mean rTE across participants, indicating that participants significantly underestimated the elapsed time of their quiet wakefulness. The lightest gray bar is an outlier. ***c***, rTE as a function of α power: stronger α power corresponded to longer rTE. Each dot is a participant. Black line is a regression line and gray shading is 95% CI. Data are reported for magnetometers. Extended Data Figure 1-1 reports the same outcome for gradiometers. Extended Data Figure 1-2*a* illustrates the mean source estimates of α power as likely contributor of the source analysis reported here. ***d–i***, Data from two representative participants P1 and P2. ***d***, P1 (rTE = 0.27) showed a flatter distribution of power spectral densities across sensors (blue). The individual's α peak frequency (iAPF) (dashed purple line) was determined using a spectral model fit *fooof* (Donoghue et al., 2020). Extended Data Figure 1-3*a* provides iAPF as a function of rTE. ***e***, Model fit for one sensor (blue) showing the estimated 1/f slope (dashed gray), the full spectral model (red), and the iAPF (purple dashed line). ***f***, An oscillatory dynamic analysis (cycle-by-cycle; Cole and Voytek, 2019) was applied to the same sensors to detect and quantify the α burstiness over time (green). ***g–i***, The same characterization of spontaneous oscillatory dynamics for a second participant P2 (rTE = 1.25). P2 shows stronger α power and α burstiness than P1. ***p < 0.001.

In the quiet wakefulness retrospective time experiment (Fig. 1*a*), the experimenter provided participants with the following instructions before the MEG recording: “I will record your brain activity at rest. Please, refrain from moving at all times and keep your eyes open. To help attenuate eye movements, we suggest you fixate on the black screen in front of you.” Following these instructions, the experimenter left the MEG room and waited for participants to state they were ready to start. Unbeknownst to participants, the recordings lasted 2, 4, or 5 min. From the participant's viewpoint, the recording unfolded as follows: the French word début (“start”) appeared on the screen for 1 s followed by a black screen lasting 4 s. A red dot centered on the screen appeared for 500 ms after which the screen remained black for 2, 4, or 5 min. A second red dot appeared on the screen for 500 ms at the end of the experiment. At the end of the MEG recording, the participant was immediately asked to provide a verbal estimate of how much time had elapsed between the two red dots (retrospective time estimate; rTE). In the retrospective time task, this instruction was fully unexpected by participants, as confirmed by informal debriefing following the recording.

In the quiet wakefulness prospective time task, participants were informed before the MEG recording that they would be asked to provide an estimation of how much time had passed between the two red dots (prospective time estimate; pTE). These recordings lasted 2 or 4 min.

In the retrospective time task following a visual counting task, 17 small white visual annulus were presented in the center of the screen for 120 ms each. The interstimulus interval varied pseudo-randomly between 7 and 45 s. For instance, one sequence of interstimulus-interval would be: 16, 5, 13, 22, 8, 5, 39, 1, 6, 7, 15, 7, 45, 6, 22, and 7 s. This task lasted 4 min. Participants were instructed to detect and count the random occurrences of the annulus and to report their final count at the end of the recording. The task started and ended with the same red dots, which were used as instructions to the participants in defining the retrospective duration (rTE) they were also asked to estimate at the end of the task.

Before the MEG recordings, participants' impulsiveness (psychological trait measure) was assessed using the French validated BIS-11 (Stanford et al., 2009). A total of 37 (out of 56 participants) in the main retrospective timing task and 25 (out of 26 participants) in the visual counting task completed the questionnaire.

### Behavioral analysis

Participants' retrospective (rTE) and prospective (pTE) time estimations were computed relative to the actual clock time that had elapsed between the two red dots as the ratio between the individual's verbal report and clock time. This provided a relative (hence, unitless) measure of duration estimation allowing the comparison of the 2-, 4-, and 5-min conditions. To test whether participants significantly overestimated or underestimated the elapsed time, we performed one-sample, one-tailed *t* tests of the relative time estimates (rTE and pTE). A one-tailed paired-samples *t* test was used to compare the rTE and the pTE of the individuals (*N* = 24) who performed the retrospective and prospective timing task. The coefficients of variations (CVs) were typically computed as the standard deviation of the population divided by the means of the population. The performance in visual counting task was computed as the ratio between the count reported by participants and the 17 stimuli effectively shown on the screen.

### MEG acquisition

We used a whole head Elekta Neuromag Vector View 306 MEG system (Neuromag Elekta LTD) equipped with 102 triple sensor elements (one magnetometer and two orthogonal planar gradiometers) to record electromagnetic brain activity in a magnetically-shielded-room. The sampling frequency was 1 kHz. A high-pass filter of 0.3 Hz was applied online. Horizontal and vertical electrooculograms (EOG) and electrocardiogram (ECG) were recorded during the session. Participants' head position was measured before each block by means of four head position coils (HPI) placed over the frontal and mastoid areas.

### MEG preprocessing

Signal space separation (Taulu and Simola, 2006) was applied to decrease the impact of external noise. MEG data were notch-filtered at 50 Hz to remove the power line noise. Ocular and cardiac artefacts were corrected by rejecting independent component analysis (ICA) components computed for MEG data that most correlated with detected ECG and EOG events. All MEG recordings lasted 2, 4, or 5 min. For the great majority of the analyses, and unless otherwise specified, we used the first 2 min of each dataset so as to conduct the analysis on the full set of participants. In the visual counting task, only the MEG signals outside the evoked responses elicited by the presentation of the annuli was considered for the burst analysis. To do so, 800 ms of signals were removed following each stimulus presentation. The output signals consisted of 18 epochs of unequal length. A total duration of 226 s was used for the MEG analysis in this task.

### MEG analysis

#### Power spectrum density

The continuous resting state recordings were segmented into nonoverlapping 5-s epochs to compute the power spectrum density (PSD). The PSDs were computed using multitaper between 0.1 and 45 Hz.

##### Spontaneous α localizer

A cluster-based analysis was performed to localize the significant sensors in the α range (7–14 Hz) separately for the magnetometers and the gradiometers using the quiet wakefulness data. All subsequent analyses for all tasks were performed using these same sensors.

In the main text, we report results for the magnetometers for simplicity and refer to them as “sensors.” All outcomes of our analyses are performed in sensor space, reported for magnetometers and could be otherwise replicated for gradiometers. Replications in gradiometers are reported in Extended Data Figures 1-1 and 1-3*b*.

On a per individual basis, the 1/f trend of the PSDs was compensated for in each epoch and sensor. For this, we computed the mean PSD per sensor and normalized them by the grand mean PSD taken over all sensors. To localize sensors most sensitive to α, we ran a cluster-based permutation analysis (Maris and Oostenveld, 2007) implemented in MNE-Python (Gramfort et al., 2013) by drawing 1000 samples for the Monte Carlo approximation and using FieldTrip's default neighbor templates for the vectorview MEG system (Oostenveld et al., 2011). The randomization method identified the MEG sensors whose statistics exceeded a critical value, with neighboring sensors exceeding the critical value defining the significant cluster. The p-value was estimated based on the proportion of the randomizations exceeding the observed maximum cluster-level test statistic. The cluster-forming threshold was set to 0.0001, which was equivalent to a t-threshold of 4.2 in an experimental design using 56 participants. Only clusters with corrected *p*-values of <0.05 are reported. Robust clusters of 39 magnetometers and 71 gradiometers were found.

##### Spectral analysis and individual α peak (iAPF) detection

The FOOOF algorithm3 (version 1.0.0) was used to parameterize neural power spectra (Donoghue et al., 2020). Settings for the algorithm were as follows: the peak width limits were set to [1.0, 8.0], the maximal number of peaks was set to 6, the minimum peak height was set to 0.1, the peak threshold was set to 2.0 and the aperiodic mode was fixed. The PSDs of significant sensors were used as FOOOF algorithm input. The algorithm outputs an estimate of the individual α peak frequency (iAPF) and power. The iAPF was defined as the local maximum within the frequency range of 7–14 Hz, and averaged across significant sensors on a per individual manner (Figs. 1*d*,*e*,*g*,*h*, 3*g*, 4*b*). Hence, α power was the average periodic power at iAPF across significant sensors. The median absolute error for iAPF estimation was between 0.1 Hz for low noise and 1.25 Hz for high noise.

##### Oscillatory bursts analyses

**Figure 2.**
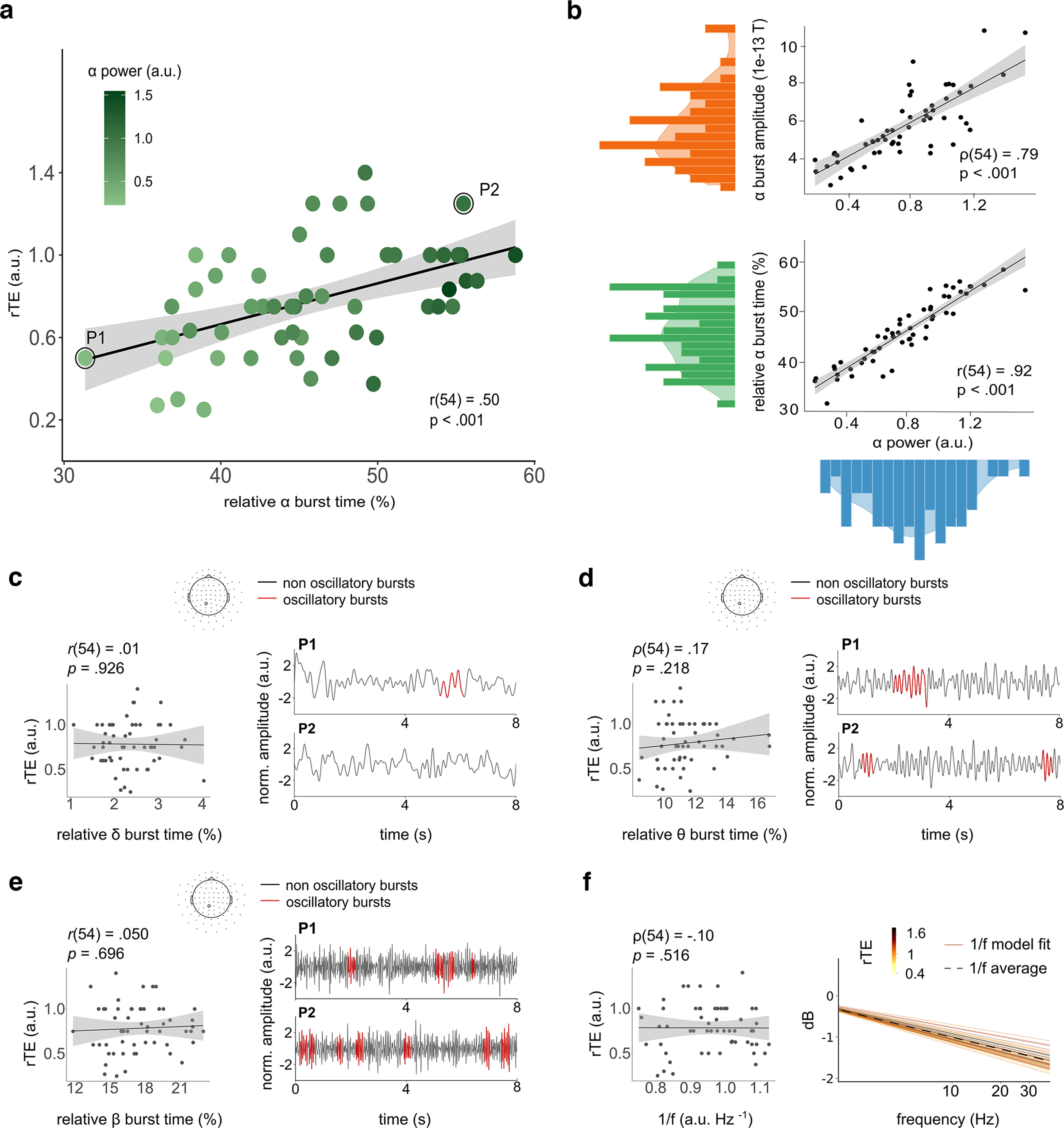
α Power and relative α burst time predict retrospective timing. ***a***, relative retrospective time estimates (rTE) as a function of the relative α burst time (%), that is, the relative amount of time α was bursting during the quiet wakefulness period participants estimated the duration of. Participants' rTE significantly increased with higher relative burst time. Each dot is a participant. P1 (rTE = 0.27) and P2 (rTE = 1.25) are two participants whose spectral dynamics are provided in Figure 1. Black line is a regression line and gray shading is 95% CI. Data are reported for magnetometers. Identical outcomes for gradiometers are provided in Extended Data Figure 1-1. ***b***, Distribution of α burst amplitude (top panel, orange) and relative α burst time (bottom panel, green) as a function of α power (blue). ***c***, Left panel, Relative δ (δ: 1–3 Hz) burst time did not correlate with rTE. Right panel: dynamic oscillatory δ analysis applied to the data of P1 and P2. ***d***, ***e***, The same analysis was applied for the θ (4–7 Hz) and β (15–30 Hz) bands. The relative θ or β burst times did not significantly correlate with rTE. ***f***, Left panel, 1/f components do not predict rTE. Right panel: comparison of the 1/f components of the average power spectrum across epochs and sensors for each participant. For this, the 1/f offset and exponent per participant were used to reconstruct the aperiodic-only spectrum (Donoghue et al., 2020). Each line shows the aperiodic spectrum of one participant. The dashed black line shows the mean aperiodic spectrum across participants. The yellow to brown shading indicates rTE. Extended Data Figure 2-1 further describes the stability of α burst dynamics over time.

**Figure 3.**
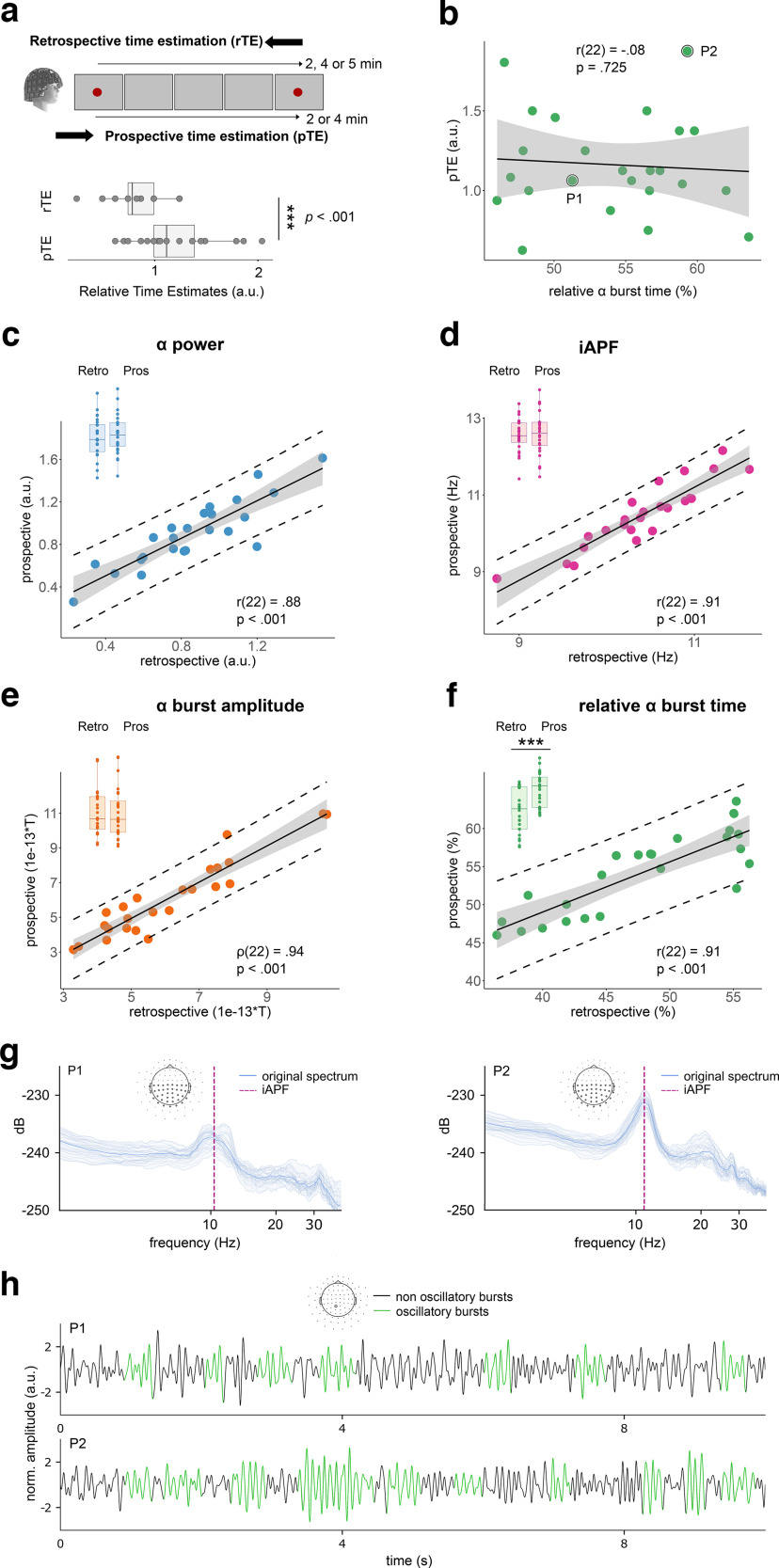
Relative α burst time does not predict prospective timing (*N* = 24). ***a***, A total of 24 participants who took part in the retrospective timing task (rTE) were now asked to estimate the duration that will elapse between the beginning and the end of the recording (prospective timing, pTE). As predicted, participants estimated the relative duration to be significantly longer in the prospective task compared with the retrospective task. ***b–f***, Each dot is a participant. Black lines are regression lines and gray shading are 95% CI. Retro is retrospective timing data; Pros is prospective timing data. ***b***, The relative duration of α burst showed no significant correlation with pTE. ***c***, α Power (blue) in prospective and retrospective tasks were significantly correlated and did not significantly differed between the two tasks (*t*_(23)_ = −1.73, *p* = .097, blue box plots). For illustration purposes, Extended Data Figure 1-2*b* illustrates the mean source estimates of α power in prospective timing. ***d***, Participants' individual's α peak frequency (iAPF) (purple) in prospective and retrospective tasks were highly correlated and did not significantly differed (*t*_(23)_ = −1.08, *p* = 0.289; purple box plots). ***e***, α burst amplitude (orange) in the two tasks significantly correlated and did no significantly differed (*t*_(23)_ = −0.05, *p* = 0.960; orange box plots). ***f***, The relative α burst time (green) in prospective and retrospective timing was strongly correlated but differed between the two tasks: the relative α burst time was significantly higher in prospective than in retrospective timing task (*t*_(23)_ = −8.80, *p* < 0.001; green plots). Data from participants P1 and P2 recorded during the prospective timing task are illustrated with (***g***) power spectra and (***h***) oscillatory dynamics. ***p < 0.001.

**Figure 4.**
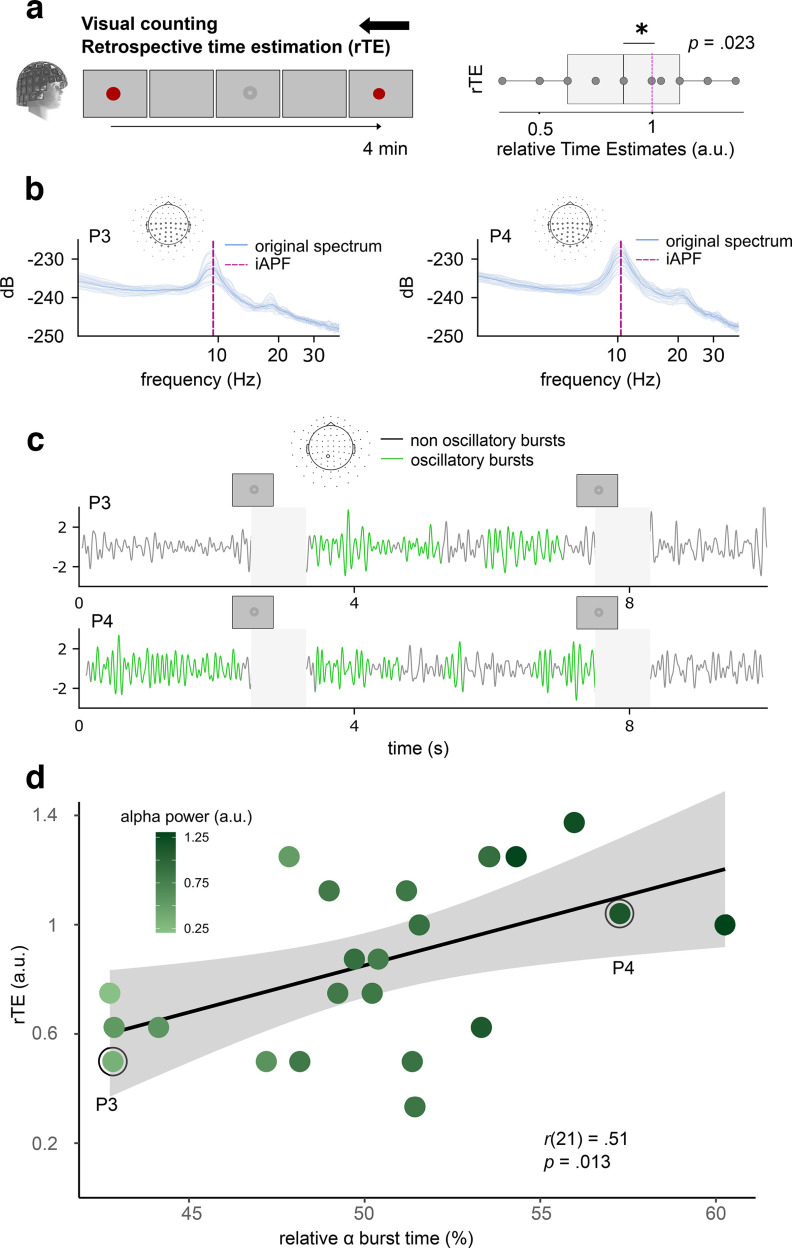
α Power and relative α burst time predict retrospective timing despite a visual counting task (*N* = 23). ***a***, Participants counted faint visual stimuli displayed at random times on the screen during the MEG recording. No instructions about timing was provided. Participants performed well on the counting task and retrospectively underestimated the elapsed time. ***b***, ***c***, Data from two participants (P3 and P4). ***b***, P3 (rTE = 0.33) showed a flatter distribution of power spectral densities across sensors (blue) as compared with P4 (rTE = 1.04). ***c***, P3 showed fewer oscillatory bursts (green) than P4. To prevent contamination from the evoked responses elicited by the presentation of visual stimuli, 800 ms were taken out of the burst analysis (shaded gray). ***d***, rTE as a function of relative α burst time (%). Participants' rTE significantly increased with higher relative burst time and stronger α power, replicating and extending our original observations. Each dot is a participant. Black lines are regression lines and gray shading are 95% CI.

The cycle-by-cycle time-domain analysis was used to detect α oscillatory bursts in the continuous MEG recordings and to quantify each oscillatory cycle amplitude (Cole and Voytek, 2019). We ran this analysis for all three tasks on a per individual basis (Figs. 1*f*,*i*, 3*h*, 4*c*). The threshold parameters used to detect episodes with bursts were as follows: amplitude fraction threshold = 0.2; amplitude consistency threshold = 0.4; period consistency threshold = 0.4; monotonicity threshold = 0.8; and minimum number of cycles = 3. The Neurodsp tool was subsequently used to quantify the relative burst time (Cole et al., 2019), a feature which indicates how bursty a signal is: 100% means the continuous data were detected as α burst during the entire time (sustained oscillatory signal) whereas 0% means that no α oscillations were found. Relative burst time and burst amplitude were computed for each selected sensors, and then averaged across sensors on a per individual basis. The same procedure was run on all other canonical frequency bands (Fig. 2*d–f*). Thresholds for the δ, θ, and β bands were set to: amplitude fraction threshold = 0.3; amplitude consistency threshold = 0.6; period consistency threshold = 0.5; and monotonicity threshold = 0.9.

##### Source estimation of α generators

For illustration purposes, we estimated the likely cortical generators of α power in the retrospective and prospective time tasks. The individuals' anatomic MRIs (aMRIs) were imported and segmented using the FreeSurfer image analysis suites (http://surfer.nmr.mgh.harvard.edu/). A one-layer boundary element model (BEM) surface was generated to constrain the forward model. Individual forward solutions (head models: 10,242 icosahedrons/hemisphere; 3.1-mm spacing) were computed using the individual BEM model constrained by the anatomic MRI (aMRI). The aMRI and the MEG were co-registered using the anatomic fiducials (nasion; preauricular points; head surface) digitized before the MEG acquisition with the MNE-Python suite (Gramfort et al., 2013). To ensure a reliable co-registration, an iterative refinement procedure was used to realign all digitized points with the individual's scalp and was manually checked. We used the noise covariance matrix from 1 min of empty room recording before the experimental session and used a linear constrained minimum variance (LCMV) beamforming (Van Veen et al., 1997) approach on the whole brain volume, which estimated the activity of each source at the i^th^ voxel for a given time window. The source estimates were then morphed into a common Freesurfer average brain (fsaverage) for subsequent group analysis.

The activity time courses for each voxel was segmented into nonoverlapping 10-s epochs to compute the power spectrum density (PSD). The PSDs were computed using multitaper between 0.1 and 20 Hz, then averaged across all epochs to obtain one PSD per voxel, per individual. Then, we compensated the 1/f trend of the PSDs of each voxel and normalized them by the grand mean PSD taken over all voxels on a per individual basis. The grand-average α source estimates across participants for retrospective and for prospective condition are presented in Extended Data Figure 1-2*a*,*b*, respectively.

##### Statistical analyses

In the retrospective time estimations analyses, the rTE, the iAPF, the α power and the relative burst time measurements were all normally distributed as assessed by Shapiro–Wilk's test (rTE *p* = 0.232, iAPF *p* = 0.903, α power *p* = 0.809, relative α burst time *p* = 0.156). However, the assumption of normality was not achieved for the α burst amplitude (*p* = 0.011). In the prospective time estimations the pTE, the iAPF, the periodic α power, the α burst amplitude, and the relative burst time were all normally distributed as assessed by Shapiro–Wilk's test (pTE *p* = 0.254, iAPF *p* = 0.980, α power *p* = 0.909, α burst amplitude *p* = 0.068, relative burst time *p* = 0.374). In the visual counting task, the rTE, the iAPF, the periodic α power, the α burst amplitude and the relative burst time were all normally distributed as assessed by Shapiro–Wilk's test (rTE *p* = 0.168, iAPF *p* = 0.499, α power *p* = 0.419, relative burst time *p* = 0.605).

For all normally distributed variables, we performed Pearson correlation (*r*). For the non-normally distributed α burst amplitude in the retrospective time task, we used Spearman correlation (ρ). For each significant correlation, we performed the Cook's distance measure to ensure the robustness of our results.

In the retrospective time analysis, we wished to clarify which of all the predictor variables (α power, α burst amplitude and α relative burst time) was best at accounting for the variability in retrospective time estimates (rTE). For this, we devised a statistical approach that was highly sensitive to the collinearity of the data. First, we orthogonalized the predictor variables using principal component analysis (PCA). Then, we performed a principal component regression (PCR) to select the best (or combination of) PCA predictor(s) explaining rTE. Last, we performed multiple linear regressions to disentangle statistically the best predictor(s) of rTE. Before applying PCA, we observed that the α burst amplitudes were not normally distributed because of two outlier values. Hence, we replaced these two values by the mean of the population: the α burst amplitude was then normally distributed as assessed by Shapiro–Wilk's test (*p* = 0.158). The initial eigenvalues indicated that PCA1 and PCA2 explained 84% and 14% of the variance, respectively. We excluded PCA3, which explained only 3% of the variance. Second, we performed a PCR using PCA1 and PCA2, which showed that PCA1 significantly predicted rTE (β = 0.08, *t*_(53)_ = 3.83, *p* < 0.001) whereas PCA2 did not (β = −0.05, *t*_(53)_ = −1.02, *p* = 0.310). Hence, we selected PCA1 for the last step. Last, we conducted four independent linear regressions using rTE as dependent variable and α power, α burst amplitude, α relative burst time and PCA1 as predictors. The goodness-of-fit of these four models were assessed using the Akaike Information Criterion (AIC; the lowest the AIC, the better the fit) from which we can conclude that the relative α burst time was the best predictor of rTE (Table 1).

**Table 1.** Model comparisons for the prediction models

Predictor	*p*-value	*F* value	β	*R*²	AIC
α Relative burst time	<0.0001***	18.09	0.50	0.25	−1.20
PCA1 (combination of α power, α burst amplitude and α relative burst time)	<0.001***	14.62	0.46	0.21	1.56
α Power	<0.001***	12.50	0.43	0.19	3.32
α Burst amplitude	0.016*	6.23	0.32	0.10	8.86

*F* values indicate whether the regression model provides a better fit to the data than a constant value. β Provides the standardized regression weights. *R* represents the zero-order correlation. The Akaike Information Criterion (AIC) was calculated for all models. A lower AIC value indicates a better fit. The α relative burst time predictor showed the best fit.

To compare the the iAPF, the periodic α power, the α burst amplitude and the relative burst time between retrospective and prospective timing task, we used paired two-sided *t* tests.

## Results

In the retrospective time task (Fig. 1*a*), participants were asked to remain in quiet wakefulness with opened eyes fixating on a screen placed in front of them while being recorded with MEG. A red dot signaled the beginning and the end of the recording, which, unbeknownst to participants, lasted 2, 4, or 5 min. At the end of the recording, participants were unexpectedly asked to estimate verbally and as precisely as possible (in minutes, seconds) how much time elapsed between the two red dots. We characterized participants' retrospective time estimations as the ratio between their reported duration and the elapsed time (clock duration) to establish a measure of relative retrospective time estimates (rTE). An rTE above 1 indicates that participants overestimated elapsed time, an rTE below 1 indicates that participants underestimated it.

10.1523/JNEUROSCI.0816-23.2023.f1-1 Extended Data Figure 1-1 α Burstiness in gradiometers. The same analysis performed in Figure 1*c* (rTE as a function of α power) and Figure 2*a* (rTE as a function of relative α burst time) was replicated with gradiometers. The spontaneous α localizer resulted in 71 gradiometers used for the α cycle-by-cycle analysis. Each dot represents an individual participant. The black line is the regression line and the gray shading is 95% CI. A significant positive correlation between α power (M = 0.76 ± 0.28 a.u.) and rTE was observed. The correlation between rTE and relative burst time (M = 45 ± 6%) was also significant. Download Figure 1-1, EPS file.

10.1523/JNEUROSCI.0816-23.2023.f1-2 Extended Data Figure 1-2 Cortical generators of α power. ***a***, The grand-average source estimates of α power collected in the main retrospective task was computed across participants (*N* = 50; 5 out of 56 participants did not have an aMRI). ***b***, The same analysis performed for the prospective task data (*N* = 22; 2 out of 24 participants did not have an aMRI). These serve as illustrations of likely α sources. All spectral dynamic analyses were otherwise carried out in sensor space. Download Figure 1-2, EPS file.

10.1523/JNEUROSCI.0816-23.2023.f1-3 Extended Data Figure 1-3 No relationship between an individual's α peak frequency (iAPF) and retrospective duration estimation (rTE). Each dot represents an individual. The black line is the regression line and the gray shading is 95% CI. ***a***, Magnetometers: no significant correlations were found between rTE and iAPF. The mean iAPF was 10.5 Hz (±0.78 Hz). ***b***, Gradiometers: no correlations between iAPF and rTE. Black lines are regression lines and shaded areas are 95% CI. Download Figure 1-3, EPS file.

On average, participants (*N* = 56) significantly underestimated the duration of their quiet wakefulness during the MEG recording (Fig. 1*b*; rTE = 0.78 ± 0.26, *t*_(55)_ = −6.1, *p* < 0.001). The underestimation of rTE strongly indicates that participants did not pay attention to time (Polti et al., 2018), as predicted by a lack of explicit orientation to time required by the experiment.

One property of duration estimation is its scalar property, in which the variance (σ) of a magnitude estimation increases with its magnitude (μ). As several durations were tested, we computed the coefficients of variation (CV=σrTEμrTE) for each and found that, as predicted by scalar timing, the CVs were comparable across durations (Gibbon, 1977; 2 min: CV = 31%, 4 min: CV = 35%, 5 min: CV = 35%), legitimizing the psychological effectiveness of the retrospective verbal estimations (Chaumon et al., 2022; Balcı et al., 2023).

Because of the known relation between impulsivity and timing (Wittmann and Paulus, 2008), we also tested the correlation between rTE and participants' impulsiveness scores (Stanford et al., 2009). We found no significant correlations (ρ_(35)_ = 0.03, *p* = 0.871) between these two measures, suggesting that rTE was selective to time estimation and did not reflect an individual's psychological trait.

We then turned to the individuals' MEG recordings to quantify α activity. We found that stronger α power during quiet wakefulness predicted larger rTE (Fig. 1*c*; Extended Data Fig. 1-1; *r*_(54)_ = 0.43, *p* < 0.001): the larger the α power, the longer the retrospective durations. Given this result, we explored iAPF (Haegens et al., 2014), which has been implicated in numerous perceptual timing experiments (Samaha and Postle, 2015; Cecere et al., 2017; Minami and Amano, 2017; Ronconi et al., 2018; Mioni et al., 2020). At the scale of minutes, and under the assumption that spontaneous α oscillations are stationary, the α clock hypothesis would have predicted a positive and linear relation between iAPFs (Fig. 1*d*,*e*,*g*,*h*) and an individual's rTE (Treisman, 1984). However, we found no evidence linking iAPF and retrospective duration estimation (*r*_(54)_ = −0.10, *p* = 0.469; Extended Data Fig. 1-3), suggesting the α clock hypothesis does not hold as originally conceived.

The novel observation that α power linearly correlates with individuals' retrospective duration estimates relied on time-averaged spectral quantifications, which reduce and impoverish the temporal structure of brain activity over minutes to a single characterization (i.e., α power). As neural oscillations show burstiness with fluctuating amplitudes, frequencies, and waveform morphologies (Cole and Voytek, 2017), we asked whether the relation between α power and rTE could be better accounted for by the dynamics of spectral fluctuations. In particular, we questioned whether the relative burstiness of α rhythms would be a major predictor of elapsing time. Using state-of-the-art analyses (Cole and Voytek, 2019), we detected the presence of α bursts in the MEG data (Fig. 1*f*,*i*), quantified their amplitude and the relative time of α bursts during the time interval participants had to estimate (Fig. 2). The relative burst time indexes the oscillatory dynamics of α activity and ranges from 0% to 100%, signifying no-to-sustained oscillatory α activity, respectively.

10.1523/JNEUROSCI.0816-23.2023.f2-1 Extended Data Figure 2-1 Stability of spontaneous α dynamics during quiet wakefulness. To test whether the properties of α dynamics showed a continuous trend in the course of the MEG recordings, we computed the α power, the iAPF, the α burst amplitude and the relative α burst time in moving windows of 30 s over the first 240 s of the quiet wakefulness recordings (*n* = 41; 4- and 5-min conditions). Each dot represents an individual participant. Nonparametric repeated measures Friedman test (non-normal distribution) were performed using time windows (8) as main factor. One boxplot is a time window. ***a***, A main effect of windows was found on α power (χ^2^_(7,40)_ = 67.3, *p* < 0.001). A pairwise Wilcoxon signed-rank test showed that α power showed initially less amplitude than in the rest of the recording (*p < 0.001) with α power reaching a plateau within 60 s (*p* = 1). ***b***, iAPF was stable throughout and did not change over time (χ^2^_(7,40)_ = 7, *p* > 0.05). α Burst amplitude (***c***) and relative burst time (***d***) were initially significantly lower than in the rest of the recordings (α burst amplitude: χ^2^_(7,40)_ = 61.7, *p < 0.001; relative α burst: χ^2^_(7,40)_ = 53.7, *p < 0.001). Download Figure 2-1, EPS file.

We found a significant positive correlation between rTE and relative α burst time (*r*_(54)_ = 0.50, *p* < 0.001; Fig. 2*a*), indicating that the relative duration estimated retrospectively could be predicted by the relative amount of α bursts in the absence of overt attention to time. This could be predicted as the mean spectral estimation of α power intuitively fluctuates with both α burst amplitude (Fig. 2*b*, upper panel) and relative α burst time (Fig. 2*b*, lower panel). Interestingly, characteristics of α spectral dynamics were stable for the entire period of quiet wakefulness (Extended Data Fig. 2-1) and although the correlation between α burst time and rTE varied over time, it remained systematically significant. In the initial 30 s, α burst time showed a significant correlation with rTE (ρ_(39)_ = 0.46, *p* < 0.002), which steadily increased until ∼120 s and (60 s: *r*_(39)_ = 0.43, *p* = 0.005; 90 s: ρ_(39)_ = 0.52, *p* < 0.001; 120 s: *r*_(39)_ = 0.58, *p* < 0.001) and then decreased (150 s: *r*_(39)_ = 0.46, *p* = 0.003; 180 s: *r*_(39)_ = 0.47, *p* = 0.002; 210 s: *r*_(39)_ = 0.37, *p* = 0.013; 240 s: *r*_(39)_ = 0.33, *p* = 0.037).

Given that the different α characterizations are highly collinear, we performed a principal component regression analysis to establish whether the relative α burst time was a better predictor of rTE than α power, α burst amplitude, or all of them combined (Table 1). The first principal component significantly predicted rTE (PCA1: β = 0.08, *t*_(53)_ = 3.83, *p* < 0.001; PCA2: β = −0.05, *t*_(53)_ = −1.02, *p* = 0.310) and it was selected for the independent linear regressions using rTE as dependent variable and α power, α burst amplitude, α relative burst time and PCA1 as predictors. The goodness-of-fit of these four models were assessed using the Akaike Information Criterion (AIC; the lowest the AIC, the better the fit) from which we could conclude that the relative α burst time alone was the best predictor of rTE (Table 1).

For theoretical reasons our primary working hypothesis targeted α activity. However, because different oscillations have been reported in timing tasks (Cravo et al., 2011; Kononowicz and van Rijn, 2015; Kononowicz et al., 2019; van Wassenhove et al., 2019; Herbst et al., 2022), we performed the same analysis across multiple oscillatory bands (δ: 1–4 Hz; θ: 4–7 Hz; β: 15–30 Hz) to test the spectral selectivity of our findings. Besides α, none of the tested spectral bursts were indicative of rTE (Fig. 2*d*,*e*, δ: *r*_(54)_ = −0.01, *p* = 0.926; θ: ρ_(54)_ = 0.17, *p* = 0.218; β: *r*_(54)_ = 0.05, *p* = 0.696). As the reported activity of time cells across species spans seconds and minutes (Pastalkova et al., 2008; MacDonald et al., 2011; Kraus et al., 2015; Issa et al., 2020; Umbach et al., 2020; Aghajan et al., 2022; Cogno et al., 2022; Tsao et al., 2022), one possibility is that slow-activity building over time would contribute to time estimations. Slow-activity could be captured as slow aperiodic activity in the spectrum, therefore, we tested whether the aperiodic spectrum or slope of the 1/f spectrum, capturing the slowest dynamics in the signals, would show dependency to participants' rTE. We found no such correlations (ρ_(54)_ = −0.10, *p* = 0.516; Fig. 2*f*). Hence, neither the spectral dynamics in other oscillatory regimes, nor scale-free fluctuations showed a significant relation with rTE, supporting that α burst time may be selective to retrospective timing.

We then asked whether the relation between α burst time and retrospective time estimates would hold when participants overtly oriented their attention to time. For this, we collected a prospective timing task in a subset of participants who took part in retrospective time task (*N* = 24). We instructed them before the MEG recording to keep track of how much time elapses between the two red dots (Fig. 3*a*, top panel). We computed the ratio between participants' verbal time estimates and clock duration as relative prospective time estimates (pTE). Participants' pTE showed a significant overestimation of duration spent in quiet wakefulness (Mean (M) = 1.20 ± 0.36 a.u., *t*_(23)_ = 2.75, *p* = 0.006). This outcome was consistent with the fact that attention to time dilates its subjective duration (Brown, 1985; Fortin et al., 2007; Polti et al., 2018). A one-tailed paired samples *t* test comparing relative time estimates between the retrospective and prospective tasks showed that participants estimated prospective durations to last significantly longer than the retrospective ones (*t*_(23)_ = 4.22, *p* < 0.001; Fig. 3*a*, bottom panel). Since we subsampled the original pool of participants (*N* = 24), we replicated and verified that their rTE (retrospective task) remained predicted by α power (*r*_(22)_ = 0.45, *p* = 0.024) and by the relative α burst time (*r*_(22)_ = 0.55, *p* = 0.005). As both relations replicated, we proceeded with the identical spectral dynamic analyses and found no significant correlations between pTE and α power (*r*_(22)_ = −0.23, *p* = 0.270) or between pTE and relative α burst time (*r*_(22)_ = −0.08, *p* = 0.726; Fig. 3*b*). Remarkably, the α oscillatory dynamics during quiet wakefulness were overall very similar whether participants were tested in the absence of attention to time instructions (retrospective) or with overt instructions to time (prospective timing) to the exception of the relative α burst time (Fig. 3*d*). This further demonstrated the selectivity of the effect in that the relative burst time of α dynamics predicted retrospective timing (rTE) but not prospective timing (pTE).

Last, we wondered whether the relation between α burst time and rTE would hold when participants were engaged in a nontiming task instead of being in quiet wakefulness. To test this, we ran another experiment in which naive participants (*N* = 23) had to count the total number of faint visual stimuli (a total of 17 events) presented on the screen during the MEG recording (Fig. 4*a*). At the end of the recording, participants were asked to report how many stimuli were detected but also, and unexpectedly for them, to report how much time elapsed between the two red dots. This experiment provides a very stringent control by emulating a more ecologically valid situation in which individuals vacate to occupations distinct from attending to time. Importantly, counting is also known to alter timing (Gaudreault and Fortin, 2013) and α activity is strongly modulated by visual attention (Hanslmayr et al., 2011; Nobre and Van Ede, 2018). Thus, this control task altered both a cognitive and a neurophysiological factor largely predicted to affect timing. On average, participants successfully reported the number of visual events (percent correct count = 0.99 ± 0.11). As we predicted, participants underestimated the duration of the task (rTE = 0.86 ± 0.31, *t*_(22)_ = −2.11, *p* = 0.023; Fig. 4*a*). We then asked whether their rTE could be predicted by α power, which was the case (*r*_(21)_ = 0.45, *p* = 0.031). We then replicated the relation between rTE and the relative α burst time (*r*_(21)_ = 0.51, *p* = 0.013; Fig. 4*d*). These results suggest that despite participants being engaged in a visual counting task, the relative burst time of α dynamics predicted individuals' retrospective timing.

## Discussion

In this series of experiments, we asked whether dynamic features of spontaneous oscillatory activity can tell time at the scale of minutes. We explored this question when individuals did not orient their attention to time (quiet wakefulness; retrospective time estimation and episodic time stricto sensu) or when they were asked to estimate time in advance (prospective time estimation). We report that the relative time of spontaneous α bursting activity during quiet wakefulness and during a visual task is a high predictor of participants' retrospective duration estimates at the scale of minutes. This relation did not hold for prospective timing, in which participants were explicitly told to pay attention to time. Our results suggest that spontaneous mechanisms keeping track of time when the observer is not told to keep track of it (retrospective) may largely differ from those used when the observer intently keeps track of it (prospective).

Out of the original studies testing the α clock hypothesis and failing to find a direct link with duration perception at the scale of minutes (Treisman, 1984; Kononowicz and van Wassenhove, 2016; van Wassenhove et al., 2019), the early study of Werboff (Werboff, 1962) stands out as being the closest to the current experimental venue. In his study, the author compared the “α wave-count” as the percentage of time α was present in the EEG signal: individuals with a lower occurrence of α waves underestimated elapsed time as compared with individuals with more α waves. However, participants were tested at a time scale of a few seconds (2 and 8 s) with a prospective time task. The methodological standards in 1962 are quite remote from our contemporary ones, making it hard to make a direct comparison with our observations. In fact, like a majority of early empirical efforts using prospective timing (for review, see van Wassenhove et al., 2019), we failed to find direct evidence between spontaneous α rhythms and prospective duration estimation. Attending to time may hinder our ability to capture the endogenous dynamics of an internal clocking mechanism because of the diversity of cognitive strategies deployed by participants to keep track of it. Indeed, a great majority of studies use prospective timing tasks, in which participants pay attention to the temporal statistics of upcoming stimuli (Grondin, 2010; Vatakis et al., 2018; van Wassenhove et al., 2019) engage oscillatory activity for a diversity of sensorimotor and cognitive factors. These may confound processes that are selective to the representation of time per se. The retrospective timing tasks used here could be argued to relate to implicit timing (Tsao et al., 2022; Sawatani et al., 2023). Implicit timing tasks typically explore subsecond-to-second temporal scales (Nobre et al., 2007; Nobre and Van Ede, 2018), which are crucial for the structuring of sensory information in perception and temporal expectations. Here, we explored the time scale of minutes and used a single-trial approach to ensure that participants were not aware of the goal of our study. Thus, no (implicit) temporal learning could take place in this experiment. Our approach is important for time scales that are most relevant to episodic timing and that last several seconds, minutes, or hours. Here, we thus used a minimalist retrospective time task and the implication of α rhythms in episodic time tracking became quite salient.

Although our results demonstrate the implication of rhythmic-like activity in episodic timing, we do not interpret these findings as evidence for a direct implementation of the α clock hypothesis, at least not in the manner it was initially formulated. Rather, and consistent with an information-theoretic view of time estimation (Hicks et al., 1976; Gallistel, 1990), we suggest that the retrospective estimation of the passage of time by participants is linked to episodic memory (Michon, 1975; Block, 1985; MacDonald, 2014) and implemented as a count of bouts of awareness (or “events”) during quiet wakefulness. The α clock hypothesis presented here is not about counting time per se; rather, it is about counting events spontaneously and endogenously instantiated as α burst. It is important that we do not interpret such counting mechanism as an explicit and overt counting process, but as an automatic parsing and time-stamping mechanism of internal events. Such episodic parsing would be most similar to an information theoretic event-based clock model (Gallistel, 1990), which can be reconciled with a symbolic approach of timing in memory (Friedman, 1993) and the possible spontaneous dynamics of time cells observed in various species (Pastalkova et al., 2008; MacDonald et al., 2011; Kraus et al., 2015; Issa et al., 2020; Umbach et al., 2020; Aghajan et al., 2022; Cogno et al., 2022; Tsao et al., 2022). This hypothesis, aligns well with a recent proposal (Tsao et al., 2022), in that α bursts may instantiate state-dependent network trajectories ultimately feeding episodic time estimation.

During quiet wakefulness, the implication of α rhythms in the regulation of the default-mode network is expected. In combined EEG and fMRI recordings, the coexistence of positive and negative fluctuations of neural networks activity with changes in α synchronization have been reported (Goldman et al., 2002; Laufs et al., 2003; Mantini et al., 2007): increases in α power tend to correlate with an increase BOLD response in thalamic and insular cortices, whereas a decrease in α power co-occurs with a decrease in occipital and frontal regions (Goldman et al., 2002; Laufs et al., 2003). Out of six resting-state networks identified during quiet wakefulness (Mantini et al., 2007), the default mode network (Raichle, 2015) and the dorsal attentional network (Fiebelkorn and Kastner, 2020) have shown significant congruence with α power fluctuations (Mantini et al., 2007). If the dorsal attention network (Fiebelkorn and Kastner, 2020) is most readily associated with the functional regulation of visual processing during perception, the default mode network (Raichle, 2015) is mostly involved in endogenous processing. While thalamo-cortical circuitries are important contributors to α activity (Steriade et al., 1990; Steriade, 1999; Halgren et al., 2019), a significant implication of hippocampal activity has been reported (Raichle, 2015). The presence of α bursts suggest that recurrent state-dependent networks may mediate transient or discrete bursts of neural firing in this frequency range. Consistent with this, α rhythms are coupled to the functional state of the default-mode network (Brookes et al., 2011) and α bursts have recently been associated with memory replay (Higgins et al., 2021). Consistent with the α clock hypothesis as an event-based episodic tracking mechanism, a recent study demonstrated that in the absence of sensory stimulation and feedforward inputs, α activity endogenously regulates spontaneous thoughts from which high level conscious features can be decoded including the where and what content (Xie et al., 2020).

While α rhythms are the earliest described oscillations in human brain activity (Berger, 1935), they are notoriously difficult to classify in the taxonomy of neural oscillations drawn from animal neurophysiology (Buzsáki and Draguhn, 2004; Buzsáki et al., 2013). While α rhythms are sometimes compared with θ oscillations seen in rodents, human θ and α rhythms show intriguingly divergent developmental trajectories (Cellier et al., 2021) with the precedence of θ rhythms incrementally dominated by α rhythms at seven to eight years old. In light of our findings, it would be particularly interesting to explore how developmental trajectories of episodic timing may or not follow those predicted by neurophysiology. Additionally, the iAPF increases with age to reach a value stable in adulthood and decreases again in aging (Lindsley, 1939; Scally et al., 2018; Cellier et al., 2021). We did not observe a correlational implication of iAPF in this study, but exploiting a larger range of iAPF across ages or longitudinally may provide reliable insights.

Taken together, we propose that a large-scale endogenous regulation of α burst activity may contribute to the internal counting of events and bouts of conscious moments, which may support time keeping mechanisms for the individual's episodic when. Given the simplicity of our experimental protocol, we believe that this novel α clock hypothesis could be tested in a large range of healthy and clinical population and could provide a neural marker for the passage of time. We interpret our findings as suggesting that in the absence of attention to time and temporal task demands, α bursts may embody discrete states of awareness like timestamps in our episodic landscape, from which accurate duration estimates can be recollected retrospectively, in the individual's future.
